# Evaluation of the relationships between physico-chemical parameters and the abundance of *Vibrio* spp. in blue crabs (*Callinectes sapidus*) and seawater from the Maryland Coastal Bays

**DOI:** 10.3389/fmicb.2024.1459077

**Published:** 2024-10-16

**Authors:** Jasmine Smalls, John Jacobs, Howard Townsend, Paulinus Chigbu, Salina Parveen

**Affiliations:** ^1^Department of Agriculture, Food, and Resource Sciences, University of Maryland Eastern Shore, Princess Anne, MD, United States; ^2^NOAA/NOS/NCCOS, Cooperative Oxford Laboratory, Oxford, MD, United States; ^3^NOAA/NMFS/ST/Ecosystems, Cooperative Oxford Laboratory, Oxford, MD, United States; ^4^Department of Natural Sciences, University of Maryland Eastern Shore, Princess Anne, MD, United States

**Keywords:** crabs, *Vibrio parahaemolyticus*, *Vibrio vulnificus*, predictive modeling, physicochemical parameters

## Abstract

**Introduction:**

Fluctuations in water quality characteristics influence the productivity of blue crabs (*Callinectes sapidus*), and the risk of human exposure to pathogenic *Vibrio* species. Thus, this study assessed the prevalence of total and pathogenic/clinical markers of *Vibrio parahaemolyticus* and *Vibrio vulnificus* in blue crabs and seawater from the Maryland Coastal Bays (MCBs) and the correlation between *Vibrio* levels and physicochemical parameters.

**Methods:**

Three to five crabs and 1 L of seawater were collected monthly for 3 years (May 2018 to December 2020) from six sites within the MCBs. Hemolymph and crab tissue were extracted and pooled for each site. Extracted hemolymph, crab tissue, and seawater were analyzed for *V. parahaemolyticus* and *V. vulnificus* using the Most Probable Number (MPN) and real-time PCR methods. A one-way Analysis of Variance (ANOVA), correlations, and linear models were used to analyze the data. Akaike Information Criterion (AICc) was evaluated to determine the model that provides the best fit to the data relating to *Vibrio* concentrations and environmental factors.

**Results:**

Results suggested that environmental factors could influence the growth of *Vibrio* spp. Both *V. parahaemolyticus* and *V. vulnificus* were more prevalent during the warmer months than colder months. *Vibrio* was more prevalent in crab samples compared to seawater. *Vibrio vulnificus* concentrations in seawater and hemolymph were positively correlated with temperature (*p* = 0.0143 seawater) and pH (*p* = 0.006 hemolymph). A negative correlation was observed between the concentration of *V. vulnificus* in whole crab (tissue) and dissolved oxygen level (*p* = 0.0256). The concentration of *V. parahaemolyticus* in seawater was positively correlated with temperature (*p* = 0.009) and negatively correlated with dissolved oxygen (*p* = 0.012).

**Discussion:**

These results provide current information on the spatial and temporal distributions of *Vibrio* spp. in the MCBs that are useful for implementing more efficient processing and handling procedures of seafood products.

## Introduction

The blue crab (*Callinectes sapidus*) is a crustacean species found in the coastal ocean and estuaries. At specific points in their life cycle, they can become vulnerable to parasitic dinoflagellate, viral and bacterial infections. Specifically, juvenile crabs are reported to be susceptible to pathogens including dinoflagellate parasites, such as *Hematodinium* spp., during the Fall season (Lycett et al., 2018). An optimal environment for this crab species is coastal embayment’s or areas with differing bottom structures that are influenced by both strong water circulation and tides. These environmental conditions are necessary for ensuring the productivity and longevity of the blue crab (Hines et al., 2003). Blue crabs are a very vital crustacean species for numerous reasons. They are economically important because of the high revenue they generate, including in the Chesapeake Bay and Maryland Coastal Bays (MCBs) regions (NOAA, 2022). Moreover, blue crabs are essential for the productivity and stability of many estuarine ecosystems due to their ability to serve as both a predator and prey (Baird and Ulanowicz, 1989; Seitz et al., 2005). Furthermore, given that blue crabs are opportunistic or bottom feeders they can accumulate pathogenic bacteria such as *Vibrio* spp. in their tissues that can be harmful to humans and decrease the revenue from seafood products (Zohar et al., 2008; Froelich and Noble, 2016; Campbell et al., 2022).

*Vibrio* spp. are microorganisms that naturally exist in marine environments (Baker-Austin et al., 2018; Kumarage et al., 2022). They are abundant and thrive in warm water conditions with high salinity concentrations. Humans are more likely to acquire *Vibrio-*related infections during the warmer months, which may occur by exposing an existing or new wound to contaminated water, or by ingesting undercooked or raw seafood. In the Unites States (U.S.), such infections account for 65% of seafood-borne related illnesses (Newton et al., 2012; Sampaio et al., 2022). Furthermore, as seafood consumption increases, so does the risk of human infections from exposure to pathogenic *Vibrio* spp. (Baker-Austin et al., 2024). Fishermen and seafood handlers have a higher risk of contracting such *Vibrio-*related infections because of their close contact with potential *Vibrio* spp. contaminants.

The main non-cholera *Vibrio* species that are of concern are *Vibrio parahaemolyticus* and *Vibrio vulnificus*, given that they cause seafood and water-borne related diseases in humans (Baker-Austin et al., 2018). *Vibrio parahaemolyticus* is the leading cause of gastroenteritis, with symptoms that include nausea, vomiting, and diarrhea, along with abdominal cramps and pain (Letchumanan et al., 2019). Many cases of gastroenteritis possibly go unreported because observed symptoms are not severe enough to necessitate medical attention (Drake et al., 2007). A recently documented outbreak of *V. parahaemolyticus* was due to the importation of crabmeat from Venezuela (Seelman et al., 2023). This outbreak resulted in 26 illnesses and nine hospitalizations (CDC, 2018). Illnesses caused by *V. vulnificus* are rare but can be fatal if contracted (Wang et al., 2020). For example, when *V. vulnificus* invades a preexisting wound, it may cause septicemia (Coerdt and Khachemoune, 2021), which can possibly result in death. A *V. vulnificus* outbreak occurred along the eastern region of the U.S. during the summer of 2023, which resulted in five deaths after individuals were exposed to contaminated seafood and water (CDC, 2023).

Changes in environmental conditions can cause opportunistic bacterial pathogens such as *Vibrio* spp. to increase in number and accumulate in tissues and hemolymph of shellfish (Destoumieux-Garzón et al., 2020). Therefore, this study sought to investigate the relationship between the abundance of *Vibrio* spp. and physicochemical parameters within the MCBs. The purpose of this study was to: (1) determine the abundance of total (*tlh*^+^ and *vvhA*^+^) and pathogenic/clinical (*tdh^+^, trh^+^,* and *v cgC^+^*-type) genetic markers of *V. parahaemolyticus* and *V. vulnificus* in blue crabs and seawater; (2) assess the association between *Vibrio* spp. levels and selected physicochemical parameters (i.e., temperature, pH, dissolved oxygen, and salinity); and (3) develop predictive models of *Vibrio* spp. levels using selected physicochemical parameters.

## Materials and methods

### Study area

**Figure 1:**
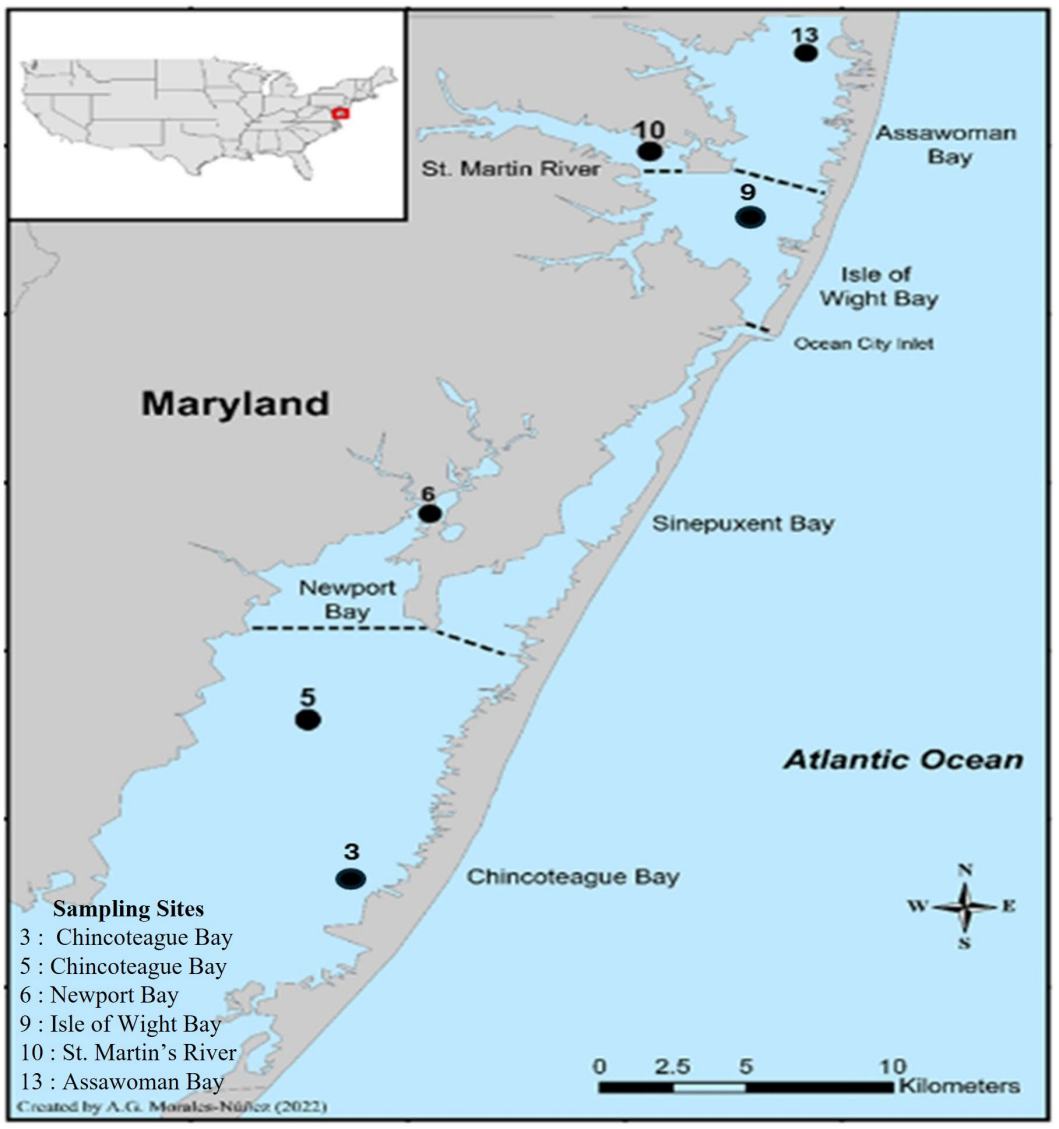
Location of the six sampling sites in the Maryland Coastal Bays.

A three-year study was conducted to gain a better understanding of *V. parahaemolyticus* and *V. vulnificus* abundance in crabs and their surrounding aquatic environment in relation to physicochemical parameters. For this study, blue crabs and seawater samples were collected from six sites: site 3 (Chincoteague lower region), site 5 (Chincoteague mid-region), site 6 (Newport Bay), site 9 (Isle of Wight Bay), site 10 (St. Martin River) and site 13 (Assawoman Bay) of the MCBs system, Maryland, U.S. (Figure 1). Sampling sites were selected based on salinity, the historical prevalence of *Vibrio* spp., accessibility, and availability of crabs (Parveen et al., 2008, 2020; Rodgers et al., 2014). The MCBs watersheds expand across several cities including Berlin, Ocean City, Pocomoke, and Snow Hill. This span allows for the geographical makeup of the MCBs to consist of several bays including Assawoman Bay, Chincoteague Bay, Isle of Wight Bay, St. Martin River, Newport Bay, and Sinepuxent Bay.

### Sample collection

A total of 36 samples (30 crabs and 6 seawater samples) were collected monthly from the six sites from May 2018 through December 2020. During each sampling event (month), 5 juvenile crabs, both males and females included (7–14 cm, carapace width), and 1 L of surrounding surface seawater were collected from each site (*n* = 6 samples per site; 5 crabs +1 L seawater). During 2020, samples were not collected until July because of the COVID-19 global pandemic quarantine mandate. Live crabs were collected using an otter trawl net, where the net was towed along the bottom of the bay at 2.5 knots for 6 min. Water samples were collected just below the surface using a Masterflex P/S water sampler system (Masterflex, Veron Hills, IL) and transferred to a sterile 1 L polyethylene bottle. After collection, crabs were bagged and chilled in an insulated chest with an ice pack and covered with a sheet of bubble wrap to prevent direct contact of crabs with the ice pack and water. The temperature was monitored using a data logger to verify that the temperature was less than 10°C during the transportation of the live crabs.

During the collection of samples, seawater temperature, salinity, dissolved oxygen, and pH were measured near the surface of the water using a portable multiparameter meter (YSI, Yellow Springs, OH, USA). Water quality parameters were recorded as previously described by Rodgers et al. (2014). These water quality parameters were chosen because (1) temperature is known to have a direct impact on *Vibrio* growth rates; (2) salinity can influence *Vibrio* metabolic and reproduction rates; (3) dissolved oxygen can affect *Vibrio* respiratory mechanisms; (4) and pH can be altered by other environmental factors, which can inhibit or promote *Vibrio* growth. Both seawater and crab samples were processed within 24 h of collection and analyzed for the enumeration of *V. parahaemolyticus* and *V. vulnificus* using the Food and Drug Administration Bacteriological Analytical Manual (FDA-BAM, Silver Springs, MD, USA) 3-tube Most- probable number (MPN) protocols detailed below (Kaysner et al., 2004).

### Sample preparation and bacteriological analyses

#### Hemolymph extraction

Hemolymph was collected from crabs and harvested samples were examined during all three sampling years. Crabs were bled aseptically by cardiac puncture through the intersegmental membrane between the posterior of the carapace and the abdomen using a sterile 1 mL surgical syringe (18G × 1, ½ in) (Tubiash et al., 1975). The puncture site was disinfected with 70% alcohol. During each sampling period, a total of 4 mL of hemolymph was obtained from crabs collected from the same sampling site and combined, with 3 mL allocated for use in the three tubes MPN assays (1 mL of hemolymph per tube). Serial dilutions ranging from 10^−1^ to 10^−8^ were created by mixing the remaining 1 mL of hemolymph with 9 mL of phosphate-buffered saline (PBS) to establish the 10^−1^ dilution, which 1 mL was then subsequently inoculated into three tubes containing 9 mL of Alkaline Peptone Water (APW). Following inoculation, all tubes were incubated overnight at a temperature of 35 ± 2°C. All tubes were vortexed after each inoculation. Inoculated samples were then incubated overnight at 35 ± 2°C.

#### Crab tissue extraction

Each sample, which was comprised of all crabs obtained from the same site, was scrubbed, dissected, and pooled. The whole crab homogenate which consisted of all internal tissue, including gills and digestion organs (meat) and hemolymph (liquor) was weighed, transferred to a laboratory blender, and blended at maximum speed for 90 s using a sterile Waring blender jar (Waring, Stamford, CT, USA). First, 10 g from the crab homogenate was added to three 100 mL tubes of APW. Then, 1 g of the mixture was added to three additional 100 mL tubes of APW. Next, 25 g of the crab homogenate was placed into a sterile blender with 225 mL of PBS and blended for 90 s to make a 10^−1^ dilution. After blending, 1 mL of the crab homogenate was added directly to three tubes with 9 mL of APW and one tube with 9 mL of PBS to create a 10–^2^ dilution (Rodgers, 2013). Subsequently, serial dilutions and MPN methods were conducted using the procedure stated in the previous section.

#### Seawater processing

For enumeration of *V. parahaemolyticus* and *V. vulnificus*, from seawater a three-tube MPN was conducted, where 100 mL of seawater was inoculated into three 100 mL bottles of 2X APW using a sterile cylinder for each site. Similarly, 10 mL of seawater was inoculated into three 100 mL bottles of 1XAPW. Then, 1 mL of seawater was added to three tubes of 9 mL of APW and one 9 mL tube of PBS to obtain a 10^−1^ dilution (Rodgers, 2013). Serial dilutions and MPN methods were conducted using the procedure stated in the previous section.

#### Inoculation of plate agar media and colony preservation

Following the incubation period, all inoculated tubes were checked for turbidity. Turbid tubes were inoculated onto selective media, thiosulfate-citrate-bile salts-sucrose (TCBS) (Fisher Scientific, Pittsburgh, PA, USA) to determine presumptive *V. parahaemolyticus*, and modified cellobiose-polymyxin B- colistin (mCPC) agar (Fisher Scientific, Pittsburgh, PA, USA) for *V. vulnificus* using the streaking method. A sterile 3-mm loop (Fisher Scientific, Pittsburgh, PA, USA) was used to streak from 1 cm of the top of the turbid APW tubes. Inoculated plates were then incubated overnight at 35 ± 2°C. After the incubation period, the plates were examined for colony growth.

Identification of the colonies was based on their physiological properties, including cell morphology and colony pigmentation. *Vibrio parahaemolyticus* appeared as round, opaque, green, or bluish colonies on TCBS agar, while *V. vulnificus* colonies were round, flat, opaque, and yellow on mCPC plates. Three isolated colonies were selected from the positive plates and inoculated into individual wells of a 96-well plate containing APW. The plates were then incubated overnight. After incubation, 200 μL of the broth was removed for DNA isolation, and Tryptic Soy Broth (TSB) with 1% Sodium Chloride (NaCl) and 50% glycerol (Fisher Scientific, Pittsburgh, PA, USA) was added to the remaining broth in the wells. The plates were stored at −80°C for further analysis.

#### DNA isolation

The tubes containing 200 μL of APW were boiled at 100°C for 10 min using a heating block. After the samples were boiled, all tubes were immediately placed on ice for 5 min. The tubes were then centrifuged at 14,000 rpm for 2 min. Subsequently, 180 μL of the supernatant was aseptically pipetted from the surface of the tubes and placed in 96 well PCR plates. Plates were then stored and maintained at −20°C until further analysis.

#### PCR analysis

Presumptive *Vibrio* isolates were confirmed using real-time quantitative polymerase chain reaction (qPCR) assays against reference strains F11-3A for *V. parahaemolyticus* and VV-11067 for *V. vulnificus* to analyze five target genes during this study. A multiplex qPCR assay with an Internal Amplification Control (IAC) was used to confirm total (*tlh^+^*) and pathogenic (*tdh^+^* and *trh^+^*) *V. parahaemolyticus* (Nordstrom et al., 2007). A single-plex qPCR assay with an IAC was used to confirm total (*vvhA^+^*) (Panicker et al., 2004), and clinical (*vcgC^+^*-type) *V. vulnificus* (Baker-Austin et al., 2010). qPCR reactions were prepared in a 25 μL reaction volume. All reactions were performed using an ABI 7500 Real-Time PCR system (Applied Biosystems, Foster City, CA). For the detection of species-specific genes (*tlh^+^* and *vvhA^+^*), isolates were confirmed to be positive if samples amplified within 35 cycles, while 40 cycles were the threshold for pathogenic and clinical genes (*tdh^+^*, *trh^+^*, and *vcgC^+^*-type) (Nordstrom et al., 2007; Baker-Austin et al., 2010).

#### Statistical analyses

A one-way Analysis of Variance (ANOVA) was used to determine if there were any statistically significant differences between concentrations of total (*tlh^+^* and *vvhA^+^*) *Vibrio* spp. isolates among sites and sampling periods. A Chi-Square analysis was used to determine if there were any statistically significant differences between the prevalence of pathogenic and clinical (*tdh^+^, trh^+^*, and *vcgC^+^*-type) *Vibrio* spp. isolates among sites and sampling periods. Pearson’s Correlation analysis was used to assess if there was a correlation between concentrations of total (*tlh^+^* and *vvhA^+^*) and pathogenic or clinical (*tdh^+^, trh^+^*, and *vcgC^+^*-type) *Vibrio* spp. isolates and water quality parameters (temperature, salinity, dissolved oxygen, and pH). Predictive linear models were generated to determine the physicochemical factors’ influence on total (*tlh^+^* and *vvhA^+^*) *Vibrio* spp. concentrations. Generalized linear models were generated to determine the physicochemical factors’ influence on pathogenic and clinical (*tdh^+^, trh^+^, and vcgC^+^*-type) *Vibrio* spp. concentrations. For all generated models, the sampling year was used as the random variable. All generated models were generated using R Posit PBC programing statistical software version 4.2.2 (Posit PBC, Boston, MA, USA). A total of 10 different predictive models were generated and used for all total and pathogenic/clinical *Vibrio* spp. (Table 1). The best-fitted model was determined and selected by the Akaike information criterion (AICc) as the one with the lowest value (Cavanaugh and Neath, 2019). Due to the low occurrence of *Vibrio* spp. in seawater samples examined, a 1 was added to all seawater MPNs (log MPN + 1) to avoid and override any negative log MPN concentrations.

**Table 1:** Predictive variables used to determine the correlation between *Vibrio* spp. isolated from examined crab, hemolymph, and seawater samples and physicochemical parameters.

Model names	Predictive variables
TempSal	Temperature + Salinity
TempDO	Temperature + Dissolved Oxygen
TemppH	Temperature + pH
SalDO	Salinity + Dissolved Oxygen
SalpH	Salinity + pH
Temp	Temperature
Sal	Salinity
DO	Dissolved Oxygen
pH	pH
NULL	Intercept Only

## Results

### Prevalence of total (*tlh*^+^) and pathogenic (*tdh*^+^ and *trh*^+^) *Vibrio parahaemolyticus* and total (*vvhA*^+^) and clinical (*vcgC*^+^-type) *Vibrio vulnificus*

**Figure 2:**
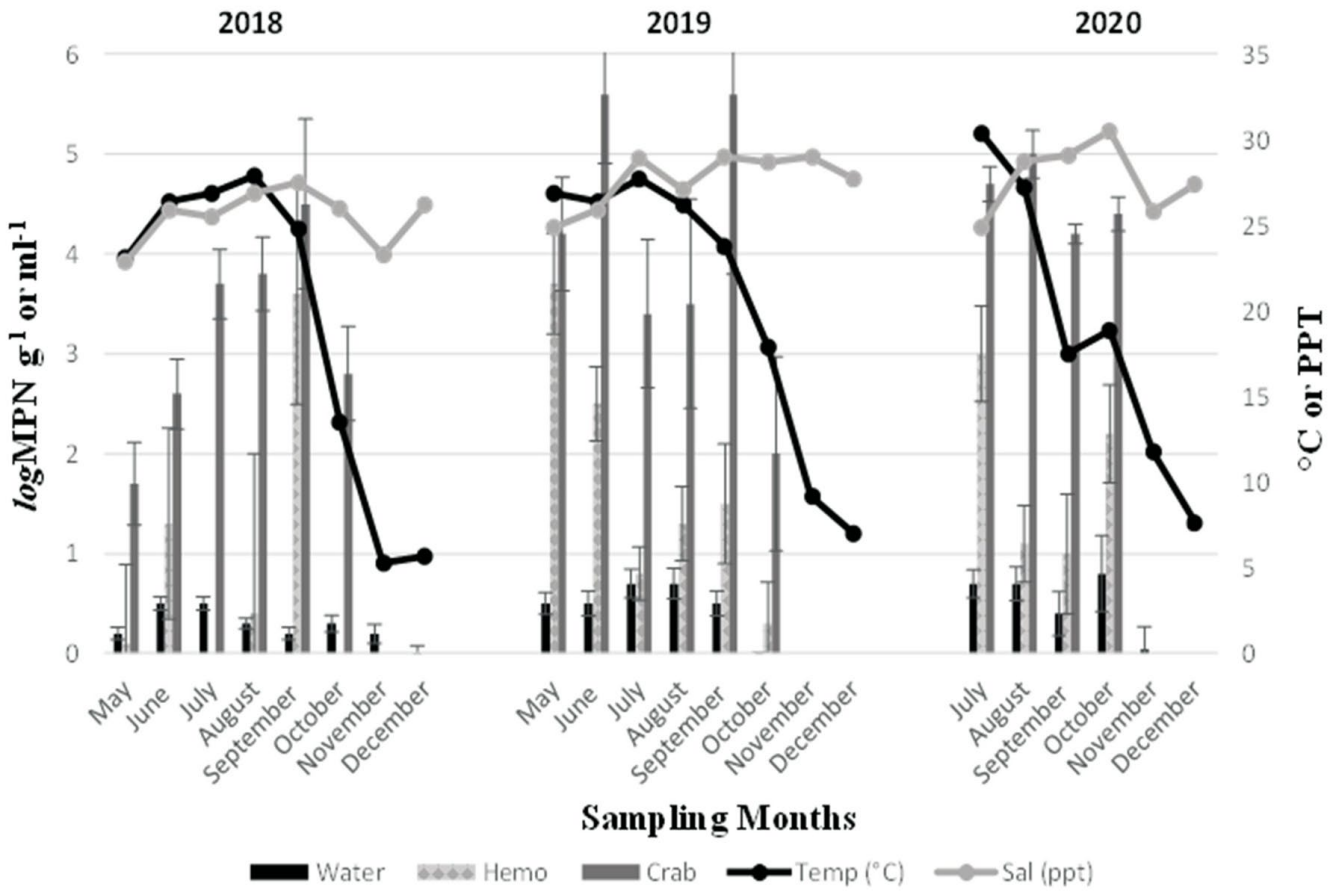
Total *Vibrio parahaemolyticus* (*tlh*^+^) in crab, hemolymph, and seawater samples for 3 years. The *x*-axis represents the months sampled. The left *y*-axis represents the average levels of total *Vibrio parahaemolyticus* for six sites. The right *y*-axis represents the average seawater temperature and salinity levels.

*Vibrio parahaemolyticus* (*tlh*^+^) was present in all crab samples examined during all three sampling years. Concentrations ranged from 1.7 to 4.5 log MPN g^−1^ (2018, Figure 2), 2 to 5.6 log MPN g^−1^ (2019) and remained above 4 log MPN g^−1^ (2020). Levels moderately increased between June and September before decreasing in October (2018) and between May and June before decreasing in July and August (2019). Also, a spike was observed in *V. parahaemolyticus* (*tlh*^+^) levels during September before declining again in October (2019). In 2020, levels were observed to be higher during July and August before slightly declining in September and October. Hemolymph was collected from crabs and harvested samples were examined during all three sampling years. Hemolymph *V. parahaemolyticus* (*tlh^+^*) concentrations ranged from 0.13 to 3.6 log MPN mL^−1^ (2018, Figure 2), 0.3 to 3.7 log MPN mL^−1^ (2019), and 1.1 to 3 log MPN mL^−1^ (2020). *Vibrio parahaemolyticus* (*tlh*^+^) was detected in 86.11% (93/108) of recovered seawater samples. Concentrations were observed to have minimal detection where concentrations remained under 1 log MPN mL^−1^ for the entire study (Figure 2). Concentrations ranged from 0.01 to 0.5 log MPN mL^−1^ (2018), 0.003 to 0.7 log MPN mL^−1^ (2019), and from 0.04 to 0.8 log MPN mL^−1^ (2020). There was a moderate increase in levels from June to July before decreasing again in August (2018). A gradual increase was observed between May and August before declining in September (2019). In 2020, levels were observed to be higher during July and August before slightly declining in September and spiking again in October.

**Figure 3:**
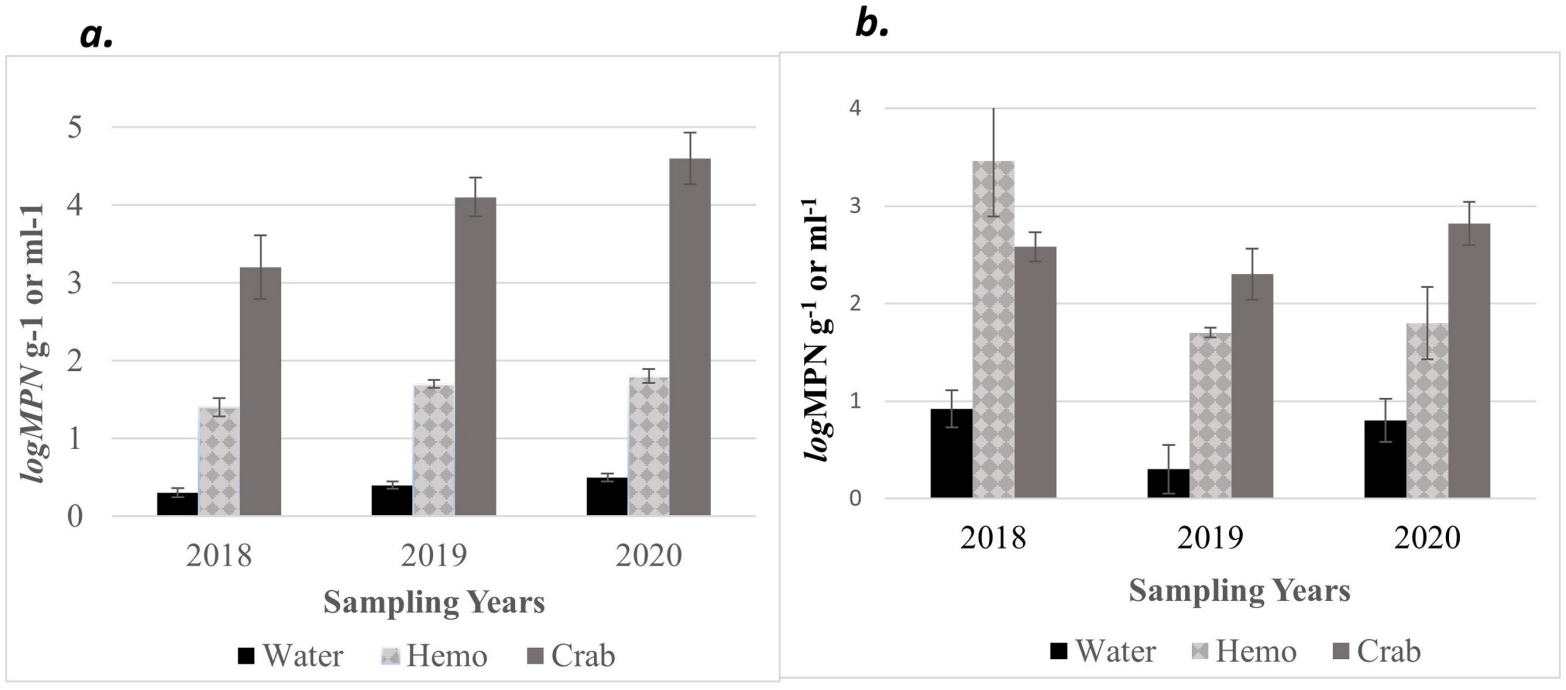
Combined annual averages of total **(a)** *Vibrio parahaemolyticus* (*tlh*^+^) and **(b)** *Vibrio vulnificus* (*vvhA*^+^) in crab, hemolymph, and seawater samples for three years. The *x*-axis represents the years sampled. The *y*-axis represents the annual average levels of total *Vibrio* spp. for six sites.

Furthermore, an annual increase was observed for *V. parahaemolyticus* (*tlh^+^*) concentrations across all sample types, where a higher prevalence of *V. parahaemolyticus* (*tlh^+^*) was detected in crabs than in seawater (Figure 3a). *Vibrio parahaemolyticus* (*tlh^+^*) levels in both crab and hemolymph samples were higher in 2020 compared to samples examined during the previous 2 years (*p* = 0.004; *p* = 0.002). There were no statistical differences found for the levels of this bacterium in either crab, or hemolymph among sites or months. *Vibrio parahaemolyticus* (*tlh^+^*) in seawater was the most prevalent during the month of July (*p* = 0.007) and had an overall higher average in 2020 (*p* = 0.002). There were no statistical differences found for the levels of this bacterium in seawater between sites. The detection of pathogenic *V. parahaemolyticus* (*tdh^+^* and *trh^+^*) was under 37% for all examined samples during 2018 and 2019 but exceeded 80% during 2020 (Table 2, *p* = < 0.001 for crab and hemolymph; *p* = 0.002 for seawater). There was no statistical difference found between pathogenic *V. parahaemolyticus* (*tdh^+^* and *trh^+^*) in crabs, hemolymph, and seawater between sites and months.

**Table 2:** Number of samples and percentages of *Vibrio* spp. genetic markers detected in examined crab, hemolymph, and seawater samples.

	Total *Vibrio* (Vp – *tlh*^+^)			Pathogenic *Vibrio* (Vp *tdh*^+^ and *trh*^+^) with percentages		
Sampling year	Seawater	Hemolymph	Crab	Seawater	Hemolymph	Crab
2018	37	10	19	10 (27.03%)	2 (20%)	5 (26.32%)
2019	43	18	19	15 (34.88%)	5 (27.78%)	7 (36.84%)
2020	28	13	18	23 (82.14%)	11 (84.62%)	15 (83.33%)

	Total *Vibrio* (Vv – *vvhA*^+^)			Clinical *Vibrio* (Vv – *vcgC*^+^) with percentages		
Sampling year	Seawater	Hemolymph	Crab	Seawater	Hemolymph	Crab
2018	22	6	14	1 (4.5%)	0 (0%)	2 (14.29%)
2019	17	6	10	2 (11.76%)	1 (16.67%)	0 (0%)
2020	14	4	9	2 (14.29%)	1 (25%)	4 (44.44%)

Analysis was made without observed statistical differences. Vp, *Vibrio parahaemolyticus*; Vv, *Vibrio vulnificus*.

**Figure 4:**
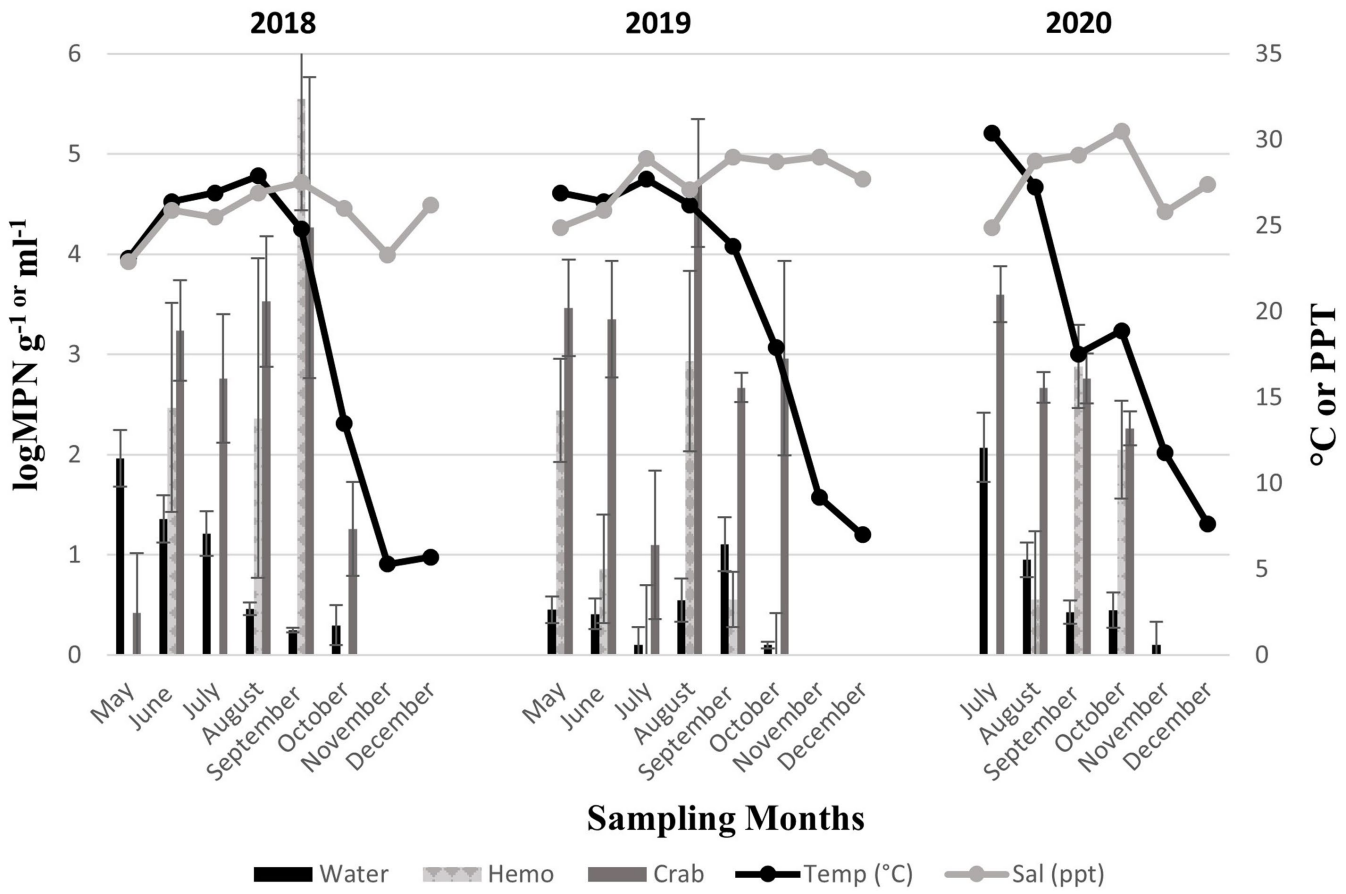
Total *Vibrio vulnificus* (*vvhA*^+^) in crab, hemolymph, and seawater samples for three years. The *x*-axis represents the months sampled. The left *y*-axis represents the average levels of total *Vibrio vulnificus* for six sites. The right *y*-axis represents the average seawater temperature and salinity levels.

*Vibrio vulnificus* (*vvhA*^+^) was present in all crab samples examined during all three sampling years. Concentrations ranged from 0.42 to 4.27 log MPN g ^−1^ (2018, Figure 4), 1.1 to 4.71 log MPN g ^−1^ (2019), and from 2.26 to 3.6 log MPN g ^−1^ (2020). Also, *V. vulnificus* (*vvhA*^+^) levels moderately increased between June and September before decreasing in October (2018). Levels remained consistent between May and June before experiencing a drastic decrease in July. Levels peaked again in August before declining once more in September (2019). In 2020, levels displayed a decreasing trend with the highest concentration of *V. vulnificus* (*vvhA*^+^) observed in July. Hemolymph was collected from crabs and harvested samples were examined during all three sampling years. The concentrations of the bacteria in the hemolymph ranged from 2.36 to 5.55 log MPN mL^−1^ (2018, Figure 4). The concentrations in the hemolymph were lower during 2019 and 2020, ranging from 0.55 to 2.93 log MPN mL^−1^ (2019) and 0.55 to 2.88 log MPN mL^−1^ (2020). In 2020, the levels displayed a drastic increase between August and September before decreasing in October. *Vibrio vulnificus* (*vvhA*^+^) was detected in 54.9% (56/102) of recovered seawater samples. Concentrations were observed to have minimal detection where concentrations remained under 2 log MPN mL ^−1^ for the entire study, except for levels in July 2020, which slightly exceeded 2 log MPN mL^−1^ (Figure 4). Concentrations ranged from 0.25 to 1.96 log MPN mL^−1^ (2018), 0.1 to 1.11 log MPN mL^−1^ (2019), and from 0.1 to 2.1 log MPN mL^−1^ (2020). Levels were observed to have an overall decreasing trend during 2018 and 2020, with detections being the highest in May (2018) and July (2020). Conversely, levels were observed to have an overall increasing trend during 2019, although, a drastic decline was detected in July and a slight peak was observed in September.

Moreover, *V. vulnificus* (*vvhA*^+^) concentrations displayed an annual decrease in all sample types between 2018 and 2019 before slightly increasing in 2020 (Figure 3b). Similarly, to *V. parahaemolyticus* (*tlh^+^*), a higher existence of *V. vulnificus* (*vvhA*^+^) was detected in crabs than in seawater. There were no statistical differences for the levels of this bacterium in either crab or hemolymph among sites, months, and years. *Vibrio vulnificus* (*vvhA*^+^) concentrations in seawater were the most prevalent in warmer months during 2018 and 2020 compared to 2019 (*p* = 0.04) and detected in higher concentrations from seawater samples collected from northern sites (*p* = 0.03). However, there were no statistical differences found in the levels of this bacterium in seawater between years. There were minimal to no occurrences observed between clinical *V. vulnificus* (*vcgC*^+^-type) in seawater and crab samples examined (Table 2). There were no statistical differences found for the levels of this bacterium in seawater or hemolymph between sites, months, and years, or crabs between sites and months. However, clinical *V. vulnificus* (*vcgC*^+^-type) levels in crabs were higher during 2020 compared to the two previous years (*p* = 0.047).

### Relationship between physicochemical parameters, and total (*tlh*^+^) and pathogenic (*tdh*^+^) *Vibrio parahaemolyticus*, and total (*vvhA*^+^) and clinical (*vcgC*^+^-type) *Vibrio vulnificus*

Environmental parameters observed during this study are outlined in Table 3. The annual mean of seawater temperatures observed were 19.2, 20.64, and 18.91°C during 2018, 2019, and 2020, respectively. The range of salinity levels during 2018, 2019, and 2020 were observed to be 22.9–27.5, 24.9–29, and 24.9–30.5 parts per thousand (ppt), respectively. Dissolved oxygen (DO) levels fluctuated throughout the year, with values ranging from 6.1–12.6, 6.8–14.9, and 5.58–11.73 mg/L during 2018, 2019, and 2020, respectively. The pH values remained consistent throughout the study during all 3 years with levels ranging between 7 and 8. The relationships between *V. parahaemolyticus* (*tlh^+^*) and physicochemical parameters are summarized in Table 4. A correlation matrix was conducted to determine those relationships. *Vibrio parahaemolyticus* (*tlh^+^*) in seawater showed a significant positive correlation with temperature and a negative correlation with DO (*p* = 0.009; *p* = 0.012).

**Table 3:** Combined averages of physicochemical parameters observed at six sites for 3 years.

Year	Month	Temperature (°C)	Salinity (ppt)	DO (mg/L)	pH
2018	May	23.1	22.9	9.0	8.1
	June	26.4	25.9	7.7	8.0
	July	26.9	25.5	6.8	7.8
	August	27.9	26.9	6.1	7.9
	September	24.8	27.5	7.3	8.5
	October	13.5	26	9.6	8.4
	November	5.3	23.3	12.6	8.1
	December	5.7	26.2	12.0	8.1
2019	May	26.9	24.9	7.1	8.0
	June	26.4	25.9	7.7	8.0
	July	27.7	28.9	6.9	7.7
	August	26.2	27.1	7.5	8.2
	September	23.8	29	6.8	7.9
	October	17.9	28.7	9.4	7.9
	November	9.2	29	12.0	8.1
	December	7	27.7	14.9	8.3
2020	July	30.37	24.89	5.58	7.6
	August	27.25	28.74	6.3	7.5
	September	17.52	29.1	8.86	7.7
	October	18.89	30.52	7.79	7.7
	November	11.8	25.8	9.7	7.9
	December	7.63	27.4	11.73	8.2

ppt, parts per thousand; DO, Dissolved Oxygen.

**Table 4:** *Vibrio* spp. relationships with physicochemical parameters using Pearson’s Correlation coefficient (r).

Sample type	Gene	*N*	Temperature	Salinity	Dissolved oxygen	pH
Crab	Vp – *tlh*^+^	56	0.03	−0.07	−0.08	−0.12
	Vp – *tdh*^+^/trh^+^	56	0.08	0.07	*−0.28	*−0.53
	Vv – *vvhA*^+^	33	0.28	−0.11	*−0.37	−0.01
	Vv – *vcgC*^+^	33	ND	ND	ND	ND
Hemolymph	Vp – *tlh*^+^	41	0.01	0.09	−0.18	−0.25
	Vp – *tdh*^+^/trh^+^	41	−0.14	0.24	−0.01	*−0.32
	Vv – *vvhA*^+^	16	0.23	−0.17	0.09	*0.65
	Vv – *vcgC*^+^	16	ND	ND	ND	ND
Seawater	Vp – *tlh*^+^	108	*0.43	0.01	*−0.41	−0.29
	Vp – *tdh*^+^/trh^+^	108	0.05	0.14	−0.1	*−0.19
	Vv – *vvhA*^+^	53	*0.36	−0.22	−0.25	−0.02
	Vv – *vcgC*^+^	53	ND	ND	ND	ND

N represents the number of samples analyzed. ND indicates that statistical analyses were not conducted due to low occurrences of the gene. An (*) denotes significance at 0.05. Vp, *Vibrio parahaemolyticus*; Vv, *Vibrio vulnificus*.

The relationship between *V. parahaemolyticus* (*tlh^+^*) levels in crab and hemolymph samples with physicochemical parameters did not generate a significant correlation. Predictive linear models were generated to determine any further correlation between levels of this bacterium and environmental factors, and the best fitted models for each sample type are outlined in Table 5. The TempDO model had the lowest AICc value from the generated *V. parahaemolyticus* (*tlh^+^*) crab model sets, indicating that temperature (Coeff. Est. = 0.17; SE = 0.09) and DO (Coeff. Est. = 1.00; SE = 0.27) had the strongest meaningful effects on *tlh^+^* levels in crab samples. The DO model had the lowest AICc value from the generated *V. parahaemolyticus* (*tlh^+^*) hemolymph model sets (Table 5), indicating that DO (Coeff. Est. = −0.34; SE = 0.18) had the strongest meaningful effect on *tlh^+^* levels in examined hemolymph samples. The TemppH model resulted in the lowest AICc value from the generated *V. parahaemolyticus* (*tlh^+^*) seawater model sets indicating that temperature (Coeff. Est. = 0.02; SE = 0.01) and pH (Coeff. Est. = 0.39; SE = 0.16) had the strongest meaningful effects on *tlh^+^* levels in seawater samples. Hemolymph was also added as a predictor to the crab models given that hemolymph is a component of the whole crab homogenate examined. Logistic regression models were generated to describe relationships between pathogenic *V. parahaemolyticus* (*tdh*^+^ and *trh*^+^) levels in environmental samples and physicochemical parameters, and the best fitted models for each sample type are outlined in Table 5. Based on the logistic regression models created, models containing salinity (crab: Coeff. Est. = 0.13; SE = 0.09, hemolymph: Coeff. Est. = 0.24; SE = 0.13, and seawater: Coeff. Est. = 0.09; SE = 0.05), and pH (crab: Coeff. Est. = − 6.04; SE = 1.57, hemolymph: Coeff. Est. = − 3.88; SE = 1.69, and seawater: Coeff. Est. = − 1.30; SE = 0.66) combined as independent variables resulted in the lowest AICc values for all three sample types examined. This indicates that salinity and pH have a major influence on the virulence factors and were the primary predictors contributing to the pathogenicity of *V. parahaemolyticus* (*tdh*^+^ and *trh*^+^) levels in all examined environmental samples.

**Table 5:** Descriptives and selection of predictive linear models used to determine the correlations between *Vibrio* spp. isolated from crab, hemolymph, and seawater and physicochemical parameters based on AICc values.

Sample type	Gene type	Predictor variables	*K*	AICc	Δ AICc	AICc weights	-2 Log likelihood	Coefficient estimates	Standard error
Crab	Vp – *tlh*^+^	Temperature + Dissolved Oxygen	5	145.88	0	0.49	−66.97	0.17	0.09
								1	0.27
	Vp – *tdh*^+^/*trh*^+^	Salinity + pH	4	76.08	0	0.43	−34.86	0.13	0.09
								−6.04	1.57
	Vv – *vvhA*^+^	Dissolved Oxygen	4	48.16	0	0.64	−17.22	−0.52	0.27
Hemolymph	Vp – *tlh*^+^	Dissolved Oxygen	3	147.54	0	0.3	−70.43	−0.34	0.18
	Vp – *tdh*^+^/*trh*^+^	Salinity + pH	4	51.1	0	0.51	−22.21	0.24	0.13
								−3.88	1.69
	Vv – *vvhA*^+^	pH	3	52.08	0	0.61	−22.04	3.48	1.08
Seawater	Vp – *tlh*^+^	Temperature + pH	4	176.01	0	0.59	−83.81	0.02	0.01
								0.39	0.16
	Vp – *tdh*^+^/*trh*^+^	Salinity + pH	3	148.54	0	0.34	−71.16	0.09	0.05
								−1.3	0.66
	Vv – *vvhA*^+^	Temperature	3	71.29	0	0.36	−32.36	0.03	0.01

The AICc value represents the best fitted model that is significant at 0.05. K represents the number of independent variables analyzed. ΔAICc represents the difference between two AICc values that are being compared. The AICc weight represents the relative likelihood between two models. −2 log (LL) is the likelihood or deviance of the outcome from the mean. Vp, *Vibrio parahaemolyticus*; Vv, *Vibrio vulnificus*.

The relationships between *V. vulnificus* (*vvhA*^+^) and physicochemical parameters are summarized in Table 4. A correlation matrix was conducted to determine those relationships. A significant negative correlation was observed between *V. vulnificus* (*vvhA^+^*) in whole crab and DO (*p* = 0.0256). A significant positive correlation was observed between *V. vulnificus* (*vvhA^+^*) in hemolymph and pH (*p* = 0.0061) and between seawater and temperature (*p* = 0.0143). General (used for total *V. vulnificus* ~ *vvhA^+^*) and generalized (used for clinical *V. vulnificus ~ vcgC*^+^-type) predictive linear models were generated to determine any further correlation between levels of this bacterium and environmental factors and the best fitted models for each sample type are outlined in Table 5. The DO model had the lowest AICc value from the generated *V. vulnificus* (*vvhA^+^*) crab model sets indicating that DO (Coeff. Est. = −0.52; SE = 0.27) had the strongest meaningful effect on *tlh^+^* levels in examined crab samples. The pH model resulted in the lowest AICc value from the generated *V. vulnificus* (*vvhA^+^*) hemolymph model sets, indicating that pH (Coeff. Est. = 3.48; SE = 1.08) had the strongest meaningful effect on *vvhA^+^* levels in hemolymph samples. The Temp model resulted in the lowest AICc value from the *V. vulnificus* (*vvhA^+^*) seawater model sets, indicating that temperature (Coeff. Est. = 0.03; SE = 0.01) had the strongest meaningful effects on *vvhA^+^* levels in seawater samples. Hemolymph was also added as a predictor to the crab models given that hemolymph is a component of the whole crab homogenate examined. Due to infrequent detection and low densities, a significant correlation and generalized linear models could not be determined between clinical *V. vulnificus* (*vcgC*^+^-type) levels in environmental samples and physicochemical parameters.

## Discussion

The crab industry has faced numerous challenges, many of which have been associated with crab’s vulnerabilities to climatic, abiotic environmental, and economic fluctuations (Apine et al., 2022). Such challenges can cause a variety of issues ranging from poor water quality (Coates and Rowley, 2022) to bacterial disease outbreaks (Tan et al., 2023). Numerous studies have previously reported the prevalence of *Vibrio* spp. in correlation to environmental factors, more specifically temperature and salinity (Rodgers et al., 2014; Siboni et al., 2016; Semenza et al., 2017; Sullivan and Neigel, 2018; Liang et al., 2019; Almuhaideb et al., 2020; Parveen et al., 2020; Wang et al., 2020; Brumfield et al., 2021; Audemard et al., 2022; Brumfield et al., 2023). Therefore, the current study aimed to investigate *V. parahaemolyticus* and *V. vulnificus* abundance in crab, hemolymph, and seawater samples over a three-year sampling period to capture environmental variability.

During this study, given the proximity of the sampling sites, little variation was found in water quality variables between sites; however, a statistically significant year to year variation was observed. For instance, coastal areas within the U.S., including the Chesapeake Bay region, experienced extreme weather conditions, such as heavy rainfall in 2018 (Chesapeake Bay Foundation, 2019). The extreme precipitation likely contributed to the increase of *Vibrio* spp. concentrations that were detected during that year allowing *Vibrio* spp. to thrive and multiply rapidly. Additionally, dual peaks in *Vibrio* levels were detected within crabs mainly for *V. parahaemolyticus* during 2019. These peaks may be attributed to underlying environmental factors and the overall physiological nature of the crabs examined. Also, due to the quarantine mandate in 2020, sample collection began during the height of *Vibrio* season (Froelich and Daines, 2020) missing the first 2 months of the sampling season, which might have contributed to the elevated *Vibrio* spp. concentrations observed during this sampling period.

*Vibrio parahaemolyticus* (*tlh^+^*) was detected in higher concentrations in whole crab samples during the warmer months for all three sampling years. These observations agree with results from previous studies that reported higher *V. parahaemolyticus* (*tlh^+^*) densities during warmer months and minimal detection during cooler months (Rodgers et al., 2014; Davis et al., 2017; Almuhaideb et al., 2020; Parveen et al., 2020). *Vibrio parahaemolyticus* (*tlh^+^*) levels in crabs observed during the current study correspond to its levels detected in Maryland oysters (*Crassostrea virginica*) (Parveen et al., 2020). Although both studies detected similar ranges, it is likely that the slight variation observed between *Vibrio* levels from the two studies is because of the different aquatic invertebrates examined. For example, oysters are filter feeders allowing them the ability to selectively accumulate pathogens in their tissues (Bruto et al., 2017) whereas crabs are bottom feeders, from which they can accumulate various bacteria. However, crabs can eliminate excess bacteria through hemolymph clotting (Salawu et al., 2016).

*Vibrio parahaemolyticus* (*tlh^+^*) levels in crab samples during this study had an annual average range of 3.3–4.5 log MPN g ^−1^, which differs between 2.5 and 2.8 log MPN g^−1^ from the levels reported by Rodgers et al. (2014). This difference may be attributed to the overall number and physiological functionality of the crab examined. Seawater samples were observed to have lower densities of *V. parahaemolyticus* (*tlh^+^*) compared to tissue samples, which agrees with the results of previous studies (Parveen et al., 2008; Takemura et al., 2014; Urquhart et al., 2016; Ozbay et al., 2018; Almuhaideb et al., 2020). The highest level of *V. parahaemolyticus* (*tlh^+^*) observed in seawater samples during this current study was 0.7 log MPN mL^−1^, with an annual average range of 0.2–0.5 log MPN mL^−1^. These findings are within the range of *V. parahaemolyticus* (*tlh^+^*) levels detected in seawater samples reported by Rodgers et al. (2014). However, levels from the previous study had a slightly higher range (0.47–2.38 log MPN mL^−1^).

During this study*, V. vulnificus* (*vvhA*^+^) densities in crabs were observed to have an average annual range of 2.3–2.7 log MPN g^−1^. These findings agree with results reported from previous studies (Rodgers et al., 2014; Sullivan and Neigel, 2018). Moreover, *V. vulnificus* (*vvhA*^+^) levels in hemolymph were observed to have a similar trend to that of *V. parahaemolyticus* (*tlh^+^*), in which minimal detection of *V. vulnificus* (*vvhA*^+^) was observed in all hemolymph samples examined. The highest level of *V. vulnificus* (*vvhA*^+^) detected in hemolymph examined was 5.4 log MPN mL ^−1^ in 2018, with an annual average range of 1.6 to 3.4 log MPN mL ^−1^. This range is consistent with the range of *V. vulnificus* (*vvhA*^+^) levels in hemolymph reported by Rodgers et al. (2014). The highest level of *V. vulnificus* (*vvhA*^+^) observed in seawater samples was 2.1 log MPN mL^−1^ during 2020, with an average annual range of 0.3–1.8 log MPN mL^−1^. This range is lower than the levels of *V. vulnificus* (*vvhA*^+^) in seawater observed by Rodgers et al. (2014). *Vibrio vulnificus* (*vvhA*^+^) requires a salinity range of 5–20 ppt for optimal growth (Randa et al., 2004; Wetz et al., 2014; Oliver, 2015; Deeb et al., 2018). As previously mentioned, low detection of *V. vulnificus* (*vvhA*^+^) in examined seawater samples is likely due to high salinity concentrations within the MCBs (>23 ppt), which are unfavorable for this bacterium.

The pathogenicity of *V. parahaemolyticus* is often determined by the detection of two virulence genes, thermostable-direct-hemolysin (*tdh^+^*) and *tdh*-related hemolysin (*trh^+^*) (Nordstrom et al., 2007). Apart from samples examined in 2020, pathogenic *V. parahaemolyticus* (*tdh*^+^ and *trh*^+^) was detected in <40% of all samples examined in 2018 and 2019 (Table 2). These findings agree with previous studies that reported low occurrences of pathogenic *V. parahaemolyticus* (*tdh*^+^ and *trh*^+^) in examined environmental samples (Parveen et al., 2008; Rodgers et al., 2014; Zhang et al., 2018; Almuhaideb et al., 2020). Furthermore, although pathogenic *V. parahaemolyticus* (*tdh*^+^ and *trh*^+^) was not frequently detected in all examined samples during the current study, *tdh*^+^ (40% = 82/205) had an overall higher occurrence than *trh*^+^ (23% = 47/205). This agrees with previous studies that also reported similar findings (Rodgers et al., 2014; Almuhaideb et al., 2020). Such findings suggest that both virulence factors may have different inhabitation requirements, in which certain conditions may be suitable for *tdh^+^* and not *trh^+^* accounting for the higher detection of *tdh*^+^. However, further research is needed to verify this finding.

The pathogenicity of *V. vulnificus* is reported to be determined by a variety of factors, including the presence of an extracellular polysaccharide capsule and endotoxins (Linkous and Oliver, 1999; Oliver, 2015). The presence of the virulence-correlated gene clinical type (*vcgC*^+^-type) has been used as an indicator for the pathogenicity of *V. vulnificus* (Baker-Austin et al., 2010). However, no specific gene marker or genotype has been identified to directly predict the virulence of *V. vulnificus* with complete certainty (Dickerson et al., 2021). During the current study, minimal to no detection of clinical *V. vulnificus* (*vcgC*^+^-type) was observed. Previous studies have observed the environmental genotype (*vcgE*^+^) to have a higher uptake and colonization rate in oysters than that of the clinical genotype (*vcgC*^+^-type) (Kim and Do Jeong, 2001; Chatzidaki-Livanis et al., 2006; Warner and Oliver, 2008; Mahmud et al., 2010; Froelich and Oliver, 2013). This may be similar for crabs given the low retention of clinical *V. vulnificus* (*vcgC*^+^-type) observed during the current study.

Extensive research has been done to examine the relationship between *V. parahaemolyticus* (*tlh*^+^) levels in seawater and temperature. The current study discovered a notable relationship between the levels of *V. parahaemolyticus* (*tlh*^+^) in seawater and temperature. This aligns with previous studies that reported similar results (Duan and Su, 2005; Parveen et al., 2008; Rodgers et al., 2014; Almuhaideb et al., 2020). Temperature was found to play a significant role in the levels *V. parahaemolyticus* (*tlh*^+^), as it is known to greatly impact the presence of these levels in estuarine environments (Duan and Su, 2005). Previous research also found that temperature was a primary factor in the most suitable models (Jacobs et al., 2015; Muhling et al., 2017; Parveen et al., 2020). Moreover, a notable inverse relationship was observed between the levels of *V. parahaemolyticus* (*tlh*^+^) in seawater and dissolved oxygen (DO) during the current study. This discovery aligns with previous studies that yielded similar results (Rodgers et al., 2014; Parveen et al., 2020). In contrast, Parveen et al. (2008) found a direct relationship between *V. parahaemolyticus* (*tlh*^+^) concentrations in seawater and dissolved oxygen (DO), indicating that DO, along with temperature and salinity, may play a crucial role in influencing *V. parahaemolyticus* (*tlh*^+^) levels. Also, pH and salinity influenced the levels of pathogenic *V. parahaemolyticus* (*tdh*^+^/*trh*^+^) during the current study. This suggests that the association between *V. parahaemolyticus*, dissolved oxygen, and pH needs to be regularly monitored and thoroughly studied.

There is limited information reported on the association between *V. vulnificus* (*vvhA*^+^) levels and dissolved oxygen in estuarine environments (Pfeffer et al., 2003). During the current study, a notable adverse relationship was detected between the levels of *V. vulnificus* (*vvhA*^+^) in crabs and dissolved oxygen (DO). This discovery aligns with findings presented by Rodgers et al. (2014). This could result from the elevated nutrient levels and subsequent presence of heterotrophic bacteria in the water column causing a decrease in oxygen levels (Carpenter et al., 1998; Rathore et al., 2016). Also, dissolved oxygen (DO) and salinity are known to have an inverse relationship. This may explain the observed negative association between *V. vulnificus* (*vvhA^+^*) levels in crabs and DO during this study. There was a notable association between levels of *V. vulnificus* (*vvhA*^+^) in hemolymph and pH, showing a positive trend. In contrast, other studies reported no connection between the levels of *V. vulnificus* (*vvhA^+^*) in the hemolymph and pH (Azandégbé et al., 2010; Rodgers et al., 2014). Findings from the current generated models suggest that *V. vulnificus* (*vvhA^+^*) does not depend only on temperature and salinity but on a combination of abiotic factors for optimal growth.

In conclusion, *Vibrio* spp. cause various infections, including gastroenteritis, necrotizing fasciitis, and septicemia (Urakawa and Rivera, 2006), rendering this bacterium of great interest. Furthermore, generating a predictive framework to understand the variability of this bacterium in crabs is important and has the potential to improve handling and processing methods to enhance safety and quality control measures. The prevention of *Vibrio*-related infections is connected to investigating the underlying factors that promote their dominance in a marine environment (Johnson et al., 2012). Findings from these investigations can furnish an evidence-based approach to mitigating the occurrence of *Vibrio*-related infections. Results from this study also suggest that blue crabs serve as a potential environmental reservoir to aid in the proliferation of *Vibrio* spp. Additionally, it facilitates the creation of appropriate harvesting and processing practices, which is to ensure decontamination and reduce pathogenic organisms in seafood products. In essence, this study provides current information on interannual variations of environmental factors that impact *Vibrio* spp. concentrations in the MCBs. However, further research is needed to gain a more coherent understanding of the potential influence of physicochemical factors on *Vibrio* spp.

## Data Availability

The original contributions presented in the study are included in the article/supplementary material, further inquiries can be directed to the corresponding author.
